# Supplementary material for: A Systematic Review of Population-Based Studies Assessing Knowledge, Attitudes, Acceptance, and Hesitancy of Pregnant and Breastfeeding Women towards the COVID-19 Vaccine
Source: Vaccines (Basel). 2023 Jul 27;11(8):1289. doi: 10.3390/vaccines11081289 (PMC10459271; doi:10.3390/vaccines11081289)
Supplement: Supplementary file 1 [file vaccines-11-01289-s001.zip › vaccines-2501871-supplementary.pdf]

Supplementary Table S1. Search strategy developed adopted in each database

Dataset	Search query	Records
PubMed/MEDLINE	"Breast Feeding"[Title/Abstract] OR "Pregnant Women"[Title/Abstract] OR "Maternal Behavior"[Title/Abstract] OR "Breast Feeding"[MeSH Terms] OR "Pregnant Women"[MeSH Terms] OR "Maternal Behavior"[MeSH Terms] OR "Postpartum Period"[MeSH Terms] OR "lactating"[Title/Abstract]<br>AND<br>("attitude"[Title/Abstract] OR "knowledge"[Title/Abstract] OR "attitudes"[Title/Abstract] OR "behaviour"[Title/Abstract] OR "hesitancy"[Title/Abstract] OR "acceptance"[Title/Abstract] OR "barrier"[Title/Abstract] OR "barriers"[Title/Abstract] OR "behavior"[Title/Abstract] OR "behaviors"[Title/Abstract] OR "behaviour"[Title/Abstract] OR "behaviours"[Title/Abstract] OR "literacy"[Title/Abstract] OR "willingness"[Title/Abstract] OR "fear"[Title/Abstract] OR "facilitator"[Title/Abstract] OR "facilitators"[Title/Abstract] OR "determinant"[Title/Abstract] OR "determinants"[Title/Abstract]) AND ("COVID-19 Vaccines"[MeSH Terms] OR "2019-nCoV Vaccine mRNA-1273"[MeSH Terms] OR "BNT162 Vaccine"[MeSH Terms] OR "COVID-19 Vaccine"[Title/Abstract] OR "COVID-19 Vaccination"[Title/Abstract] OR "COVID-19 Vaccines"[Title/Abstract] OR "COVID-19 Vaccinations"[Title/Abstract] OR "Coronavirus Vaccine"[Title/Abstract] OR "Coronavirus Vaccines"[Title/Abstract])	189
Scopus	(breast AND feeding OR lactating OR pregnant OR post AND partum OR maternal AND behavior)<br>AND<br>(attitude OR 'attitudes OR knowledge OR vaccine hesitancy OR literacy OR fear OR behaviour OR acceptance OR barrier OR barriers OR behaviour OR willingness OR facilitator OR facilitators OR determinant OR determinants) AND (sars-cov-2 vaccine OR COVID-19 Vaccine OR COVID-19 Vaccination OR COVID-19 Vaccines OR COVID-19 Vaccinations OR Coronavirus Vaccine OR Coronavirus Vaccines)	180
EMBASE	('breast feeding'/exp OR 'pregnant woman'/exp OR 'maternal behavior'/exp OR 'puerperium'/exp OR 'breast feeding':ab,ti OR 'pregnant woman':ab,ti OR 'maternal behavior':ab,ti OR 'puerperium':ab,ti)<br>AND<br>('attitude'/exp OR 'attitude':ab,ti OR 'knowledge'/exp OR 'knowledge':ab,ti OR 'vaccine hesitancy'/exp OR 'vaccine hesitancy':ab,ti OR 'literacy'/exp OR 'literacy':ab,ti OR 'fear'/exp OR 'fear':ab,ti OR ‘behaviour’:ab,ti OR ‘acceptance’:ab,ti OR ‘barrier’:ab,ti OR ‘barriers’:ab,ti OR ‘behaviour’:ab,ti OR ‘willingness’:ab,ti OR ‘facilitator’:ab,ti OR ‘facilitators’:ab,ti OR ‘determinant’:ab,ti OR ‘determinants’:ab,ti) AND ('sars-cov-2 vaccine'/exp OR 'sars-cov-2 vaccine':ab,ti OR ‘COVID-19 Vaccine’:ab,ti OR ‘COVID-19 Vaccination’:ab,ti OR ‘COVID-19 Vaccines’:ab,ti OR ‘COVID-19 Vaccinations’:ab,ti OR ‘Coronavirus Vaccine’:ab,ti OR ‘Coronavirus Vaccines’:ab,ti)	127

Supplementary Table S2. Detailed reasons for exclusion

Reason of exclusion	Number of studies	Reference
Correlation between determinants of attitude and acceptance with socio-demographic characteristics	1	Kumari, A., 2022 [18]
Aggregated data on pregnant/lactating and women with chronic conditions (data not extractable)	1	Alshahrani, 2022 [19]
Comparison between pregnant women and breastfeeding women	1	Kuciel, N., 2022 [20]

Supplementary Table S3. Main characteristics of assessed outcome(s) among the included studies, reported in alphabetical order.

Author, year [ref.]	Outcomes of interest	Outcomes definition	Tool(s) used to assess the outcomes	Number of items	Validation (yes/no)
Abuhammad, 2022 [21]	Attitude	Theoretical model including: perceived risk of contracting COVID-19, COVID-19 severity, COVID-19 vaccine benefits, barriers to receiving the COVID-19 vaccine, self-efficacy, behavioral control, trust in government, anticipated regret, knowledge, and subjective norms	APLWC	n.a.	Cronbach alpha= 0.82.
Asratie, 2022 [22]	Knowledge and attitude	Knowledge related to COVID-19 vaccination during pregnancy among pregnant women.<br><br>Attitude: Perception of risk regarding the use of COVID-19 vaccine among pregnant women	semi structured interview based on literature	59 in total	n.a.
Ceulemans, 2021 [23]	Attitude	Being willing to receive COVID-19 vaccine	7-statement 4-points Likert scale	43 in total, of which 7-item for the outcome of interest (4-likert scale) on attitude	n.a.
Ghamri, 2022 [24]	Acceptance	Being willing to receive COVID-19 vaccine	ad hoc	40 in total, of which 5-item for the outcome of interest	no, but previously used in literature
Janik, K., 2022 [25]	Acceptance	Acceptance: higher score of VAC-COVID-19 Scale	DrVac-COVID19 Scale and VAC-COVID-19 Scale	The VAC-COVID-19 scale 11-item. The DrVac-COVID19S 12-item	DrVac-COVID19 Scale Cronbach alpha= 0.86 VAC-COVID-19 Scale Cronbach alpha= 0.83
Jones, 2022 [26]	Acceptance	Having received COVID-19 vaccination	Questionnaire Health Belief Model Dimensions adapted for COVID-19	13 Likert scale items	Cronbach alpha= 0.68
Mattocks, 2022 [27]	Acceptance, Knowledge	Acceptance: Having received COVID-19 vaccination<br>Knowledge: based on VKS score	VKS and VCBS	The VKS includes 9-items, The	VCBS yes (no further details)

				VCBS is a 7-item scale	
Mhereeg, 2022 [28]	Acceptance	Having received COVID-19 vaccination	vaccination data obtained from SAIL Databank	2-open-ended questions	no
Mohan, S., 2021 [29]	Hesitancy	Having not received COVID-19 vaccination or not be willing to get vaccinated	VAX	n.a.	yes (no further details)
Obasanya, 2022 [30]	Attitude	Perceptions of COVID-19 messaging, COVID-19 preventive behaviors, vaccination intent, perceived social support and barriers, use of health and social services during COVID-19, stress, medical, and government mistrust, and demographic information.	ad hoc	55 in total	no
Preis, 2022 [31]	Acceptance	Having received COVID-19 vaccine	ad hoc	n.a.	Cronbach alpha= 0.84
Ramlawi, 2022 [32]	Acceptance	Having received at least one dose of any COVID-19 vaccine	ad hoc	79 in total	informed by review and pre-test on 552 participants
Razzaghi, Yankey, 2022 [33]	Attitude Acceptance	Attitude: vaccine concerns, importance of getting vaccinated, vaccine safety, number of family members/friends received the vaccine. Acceptance: having received COVID-19 vaccine	ad hoc	4 items developed ad hoc to assess attitude; no information regarding intention to get vaccinated	n.a.
Razzaghi, Kahn, 2022 [34]	Acceptance	Women who received a COVID-19 vaccine during pregnancy, and women who were definitely/probably getting vaccinated	ad hoc	n.a.	yes
Schaal, 2022 [35]	Hesitancy	Women who reported reasons for not receiving the vaccination	ad hoc	n.a.	n.a.
Sezerol, 2023 [36]	Hesitancy	hesitancy score calculated based on 1) benefit and protective value of the vaccine: 2) vaccine repugnance and 3) solutions for non-vaccination	Turkish vaccine hesitancy scale	12-item anti-vaccine scale (for hesitancy): 4 items about the benefit and protective value of the vaccine, 5 items about anti-	yes

				vaccination, and 3 items about the solutions for not being vaccinated	
Simmons, 2022 [37]	Hesitancy	Not intention to receive the new COVID-19 vaccine when available	ad hoc	5 items	n.a.
Skirrow, 2022 [38]	Attitude	COVID-19 vaccine willingness of pregnant women if COVID-19 vaccine availability	ad hoc	n.a.	no
Stuckelberger, 2021 [39]	Attitude	COVID-19 vaccine willingness of pregnant/breastfeeding women if COVID-19 vaccine availability	ad hoc	n.a.	n.a.
Taybeh, 2022 [40]	Attitude	n.a.	ad hoc	29, of which 4 assessing acceptance and 3 for confidence	no
Wang, 2022 [41]	Knowledge–attitude–behaviour toward COVID-19 vaccine	Willingness to receive COVID-19 vaccine	ad hoc	Knowledge of COVID-19 vaccine 7 items Attitude 10 items Behaviour 7 items	Cronbach alpha= 0.857 Factor analysis was used to evaluate the structural validity; the Kaiser-Meyer-Olkin was 0.795 and Bartlett's sphericity test reached the significance level ($p<0.001$), explaining 72.62% of the total variation

APLWC: Attitude of Pregnant and Lactating Women toward COVID-19 Vaccination Questionnaire; DrVac-COVID19S: Drivers of COVID-19 Vaccination Acceptance Scale; n.a.: not available; SAIL: Secure Anonymized Information Linkage; VAC-COVID-19: Scale to Measure the Perception of SARS-CoV-2 Vaccines Acceptance; VAX: vaccine hesitancy measurement tool; VCBS Vaccine Conspiracy Beliefs Scale; VKS: Vaccination Knowledge Scale

Supplementary Table 4. Item-by-item quality assessment for each included study, reported in alphabetical order. Using the Joanna Briggs Institute quality assessment tool.

Author, year [Ref]	Item 1	Item 2	Item 3	Item 4	Item 5	Item 6	Item 7	Item 8	Overall score	Overall appraisal
Abuhammad, 2022 [19]	2	2	-2	2	-2	-2	2	2	4	low
Asratie, 2022 [20]	2	2	-2	-1	-2	2	2	2	5	moderate
Ceulemans, 2021 [21]	2	2	-2	-1	2	2	2	2	9	moderate
Ghamri, 2022 [22]	2	2	-2	-2	-2	-2	2	2	0	low
Janik, K., 2022 [23]	2	2	-2	2	-2	-2	2	2	4	low
Jones, 2022 [24]	2	2	-2	2	-2	-2	-1	2	1	low
Mattocks, 2022 [25]	2	2	-2	2	2	2	2	2	12	high
Mhereeg, 2022 [26]	2	2	-2	-2	-2	2	-2	2	0	low
Mohan, S., 2021 [79]	2	2	-2	2	-2	-2	2	2	4	low
Obasanya, 2022 [28]	2	2	-2	-2	2	2	2	2	8	moderate
Preis, 2022 [29]	2	2	-2	2	-2	2	-1	2	5	moderate
Ramlawi, 2022 [30]	2	2	-2	2	-2	2	2	2	8	moderate
Razzaghi, Yankey, 2022 [31]	2	2	-2	-1	2	2	-1	2	6	moderate
Razzaghi, Kahn, 2022 [32]	2	2	-2	2	-2	2	2	2	8	moderate
Schaal, 2022 [33]	2	2	-2	-1	-2	-2	2	2	1	low
Sezerol, 2023 [34]	2	2	-2	2	-2	-2	2	2	4	low
Simmons, 2022 [35]	2	2	-2	-1	2	2	-1	2	6	moderate
Skirrow, 2022 [36]	2	2	-2	-2	-2	-2	-2	2	-4	low

Stuckelberger, 2021 [37]	2	2	-2	-1	-2	2	-1	2	2	low
Taybeh, 2022 [38]	2	2	-2	-2	-2	-2	-2	2	-4	low
Wang, 2022 [39]	2	2	-2	2	2	2	-1	2	9	moderate

Supplementary Table S5. Quantitative results for attitude and acceptance, along with their predictors, from the included studies, in alphabetical order. The type of effect size estimated in each included study was explicitly stated first-time appearance.

Author, year [ref,]	Attitude	Predictors of attitude	Acceptance	Predictor of acceptance
Abuhammad, 2022 [19]	mean 71± 100; range (44-100); defined as moderate	Positive attitude: Official sources of data regarding COVID-19 (β =−0.140. p=0.009); lower level of income (β =−0.141. p=0.009). and marital status (β =0.130. p=0.034).		
Asratie, 2022 [20]	negative attitude 57.3%			
Ceulemans, 2021 [21]	**Pregnant**: 61.4%;<br><br>**Breastfeeding**: 68.8%	professionally inactive: aOR 0.71 (0.57–0.89); medium educational level: 0.71 (0.62–0.83); primigravida: 1.16 (1.03–1.31)<br><br>**Breastfeeding** professionally inactive 0.69 (0.57–0.82); medium educational level: 0.68 (0.59–0.78); breastefeeding<6monts: 1.73 (1.46–2.05)		
Ghamri, 2022 [22]	68%	Gestational week OR 0.98 (0.97-0.99). higher income 0.51 (0.36-0.73). higher education level 7.20 (1.62-32.07). having had gestational diabetes mellitus 1.87 (1.14-3.05)(P=0.013). influenza vaccine recommendation during present pregnancy 4.56 (3.58-7.80). being vaccinated with tetanus vaccine during present pregnancy 1.82 (1.03-3.2). and believing that the COVİD-19 vaccine could harm their baby 0.13 (0.09-0.16)		
Janik, K, 2022 [23]			41.44% using the VAC-COVID-19 SCALE. 51.23% using the DrVAC-covid-19scale	Correlation (spearman) between negative attitude and anxiety of COVID-19 (r= 0.02) and between negative attitude and fear of COVID-19 (r= 0.08)
Jones, 2022 [24]			59%	Perceived benefits OR 1.93 (1.23-3.04); perceived barriers 0.82 (0.71-0.95); higher educational level 2.57 (1.20-5.48); received all recommended vaccines 4.65 (2.12-10.22); higher age 0.90 (0.81-0.99); minoritized race 2.84 (1.20-6.73)

Mattocks, 2022 [25]			31%	
Mhereeg, 2022 [26]			68%	Age group 30–39 years HR: 1.17 (1.11–1.23); Asian 1.12 (1.00–1.25) 0.047; lowest income: 0.88 (0.82–0.95)
Obasanya, 2022 [28]	**Pregnant** positive attitude 80%; **Maternal** positive attitude 83.4%	**Pregnat** Black women aOR: 0.23 (0.08-0.67); primigravida 2.20 (0.86-5.61); ever receiving the influenza 4.22 (1.51-11.82) **Maternal** Black women 0.52 (0.23. 1.16) ever receiving influenza 3.78 1.89. 7.55)		
Preis, 2022 [29]			51.61%	socioeconomic status. black/african ethicity. perceived threat of COVID-19. behavioural attitudes toward infection mitigation (structural equation model)
Ramlawi, 2022 [30]			50.56% pregnant; 40.98% breastfeeding	Pregnant: Spoken to HCP about vaccine during perinatal period aOR 2.65 (1.87–3.77); Understand how vaccine works 1.72 (1.28–2.30); Believe vaccines are effective 1.44 (1.01–2.07); fear of Pregnancy loss 0.54 (0.40-0.74); fear for Fetal growth and development problems 0.59 (0.43–0.89); fear for Child behavioural neurodevelopmental problems 0.62 (0.46–0.85). Not significant fear of infertility. Breastfeeding: Spoken to HCP about vaccine during perinatal period aOR 3.48 (2.61–4.63); Understand how vaccine works 1.17 (0.81–1.70); Believe vaccines are effective 2.65 (1.61–4.34); fear of Pregnancy loss 1.92 (1.06–3.49); fear for Fetal growth and development problems 0.53 (0.30–0.93). Not

				significant fear of infertility; fear for Child behavioural neurodevelopmental problems
Razzaghi, Yankey, 2022 [31]	**Pregnant** very concerned about covid-19: 22.6%; covid-19 vaccine safety (completely) 23.5%; importance of getting COVID-19 vaccine (very important): 83.5%; almost all friends/femily members vaccinated against covid: 59.5%<br><br>**Breastfeeding** very concerned about COVID-19 23.5%; COVID-19 vaccine safety (completely) 37.3%; importance of getting COVID-19 vaccine (very important): 83.3%; almost all friends/femily members vaccinated against covid: 59.3%		**pregnant**: 45.1%; **breastfeeding**: 51.5%	Pregnant age 18-24y PR 0.49 (0.54-0.82) 25-34y 0.80 (0.69-0.92): 35-49y ref. Race: non hipanic balck 0.74 (0.58-0.91); non hispanic asian 1.34 (1.00-1.68) non hispanic white ref. Educational status: high 0.79 (0.63-0.95); college 0.79 (0.64-0.93) graduate 0.81 (0.66-0.95) above college ref. income below poverty 0.63 (0.49-0.78); not insured 0.88 (0.67-1.08). comorbidities 1.05 (0.92-1.17) work/school requireme COVID-19 vaccination 1.76 (1.52-1.99); provider recommendation 1.38 (1.20-1.56); ever had covid (yes) 0.70 (0.57-0.82)<br>breastfeeding age 18-24 0.86 (0.71-1.02); 25-34 0.880 (0.78-0.97): 35-49 ref. Race: non hipanic balck 0.66 (0.50-0.82); non hispanic asian 1.46 (1.23-1.69) non hispanic white ref. Educational status: high 0.73 (0.59-0.87); college 0.92 (0.78-1.06) graduate 0.90 (0.77-1.02) above college ref. income below poverty 0.70 (0.56-0.85); not insured 0.98 (0.81-1.14). comorbidities 1.14 (0.99-1.29) work/school require COVID-19 vaccination 1.50(1.32-1.68); provider recommendation 1.62(1.43-1.81); ever had covid (yes) 0.79 (0.69-0.89)
Razzaghi, Kahn, 2022 [32]			Acceptance: 388 (21.7%) receiving a COVID-19 vaccine during pregnancy; 357 (24.0% (20.5-27.7) definitely/probably would get a	Women with a health care provider recommendation (aPR = 4.86 (3.34-7.05)); women who lived with someone with a

			vaccine	condition that could increase risk for serious medical complications of COVID-19 2.11 (1.67-2.67)); women who had received an influenza vaccination 2.35 (1.67-3.32)
Skirrow, 2022 [36]	59.20%	Ethnicity (minority) OR 2.20 (1.45-3.33); age <25y 1.13 (0.69-1.88) 25-29y 1.11 (0.84-1.45) 30-34y ref 35-39y 1.03 (0.78-1.36) >39y 0.91 (0.56-1.49); low income 1.98 (1.36-2.89)		
Stuckelberger, 2021 [37]	**Pregnant:** 29.7%<br>**Breastfeeding**: 38.6%	age>40y aOR 1.8 (1.1–3.2); high educational level 1.5 (1.1–2.0); italian as primary language 3.3 (1.4–8.0); tested COVID-19+ 3.3 (0.8–13.7); living with a 65y older 2.0 (0.7–6.1); COVID-19 negative impact on life 1.0 (0.8–1.4). flu vaccine last year 2.1 (1.5–2.8)		
Taybeh, 2022 [38]	56.2% intended to receive the third dose	medical-related degree (OR 1.62. CI 1.06-2.5. p = 0.028). income (OR 0.677. CI 0.52-0.88. p = 0.004). living residency (OR 0.44. CI 0.32-0.60. p < 0.001). knowing pregnant/lactating women previously infected with infectious microbe OR: 1.539 (1.07-2.23). commitment to immunization for children 3.01 (1.03-8.82). receiving an influenza vaccine 1.46 (1.04-2.05). and worried about infectious microbes 1.32 (1.15-1.52)		
Wang, 2022 [39]	57.6% intended to receive booster dose	no intention was associated with master's degree or above (OR=0.04 (0.01-0.42); higher anxiety scores 0.76 (0.61-0.95); higher depression scores 0.83 (0.74-0.93); mixed feeding 3.17 (2.03-4.95); had longer breastfeeding duration 1.32 (1.16-1.51); had a history of high-risk travel 3.88 (1.57-9.62); had a better physical condition 4.76 (1.49-15.20); higher COVID-19 attitude scores 1.18 (1.10-1.27)	45.8% vaccinated	

a: adjusted; β: beta coefficient; HR: hazard ratio; OR: odds ratio, p: p-value; PR: prevalence ratio; r: spearman correlation; y: years

Supplementary Table S6. Quantitative results for hesitancy, fear and knowledge, along with their predictors, from the included studies, in alphabetical order. The type of effect size estimated in each included study was explicitly stated first-time appearance.

Author, year [ref.]	Hesitancy	Predictors of hesitancy	Fear	Predictor of fear	Knowledge
Abuhammad, 2022 [19]			fear of side effects 39.7%; and being pregnant 24.2%		
Asratie, 2022 [20]			fear of COVID-19 vaccine during pregnancy= 34.2%	negative attitude aOR: 6.05 (3.88–9.43); Unplanned pregnancy 3.66(2.31–5.81); higher distance form healthcare facility: 4.57(2.34–8.91); no antenatal care use: 7.1(4.35–11.5); lower level of education: 5.18(2.17–12.4); parity (<5) 0.72(0.38–1.37); COVID-19 knowledge: 1.34(0.88–2.05)	inadequate knowledge= 53.1%
Janik, K., 2022 [23]			Fear of COVID-19 Scale FCV-19s mean value= 14.59		
Mattocks, 2022 [25]			Vaccine Conspiracy Beliefs Scale among acceptant (mean) 13.0 compared to refusal 18.6, p<0.001	Vaccine Conspiracy Beliefs Scale (aOR: 0.76; 95% CI: 0.62–0.93) predicted receipt of a	Vaccination Knowledge Scale among acceptant (mean) 7.3 compared to refusal 5.2, p=0.001. higher scores on the Vaccination Knowledge Scale (aOR: 1.66; 95% CI:

				COVID-19 vaccine	1.07–2.56) predicted higher scores on the Vaccination Knowledge Scale (aOR: 1.66; 95% CI: 1.07–2.56) predicted receipt of a COVID-19 vaccine
Mhereeg, 2022 [26]				predictor of refusing: anxiety over the decision, potential long term effects, poor research, waiting after the pregnancy	
Mohan, 2021 [27]	refusal: 25%; hesitant= 25.9%.	hesitancy was significantly increased by concern over vaccine safety (χ^2=60.2, p<0.0001), not feeling safe after vaccination (χ^2=93.0, p<0.0001), not relying on vaccination (χ^2=60.3, p<0.0001), worry about vaccine problems (χ^2=22.7, p<0.0001), natural immunity lasts longer (χ^2=14.6, p<0.01) and is better (χ^2=20.9, p<0.0001) and safer (χ^2=26.7, p<0.0001) than a vaccine.			
Razzaghi, Kahn, 2022 [32]	Common reasons for nonvaccinating included concerns about safety risk to baby (37.2%) or self (34.6%) and about rapid vaccine development (29.7%) and approval (30.9%)				
Schaal, 2022 [33]	**Pregnant** 28,5% **Breastfeeding** 28,5%	**Pregnant**: Reasons for vaccine hesitancy: scientific data are too preliminary 87.8%, too little information 60.9%,			

		anxious about unborn safety 85.9%, associated complication in pregnancy 78.4%<br>**Breastfeeding**: Reasons for vaccine hesitancy: scientific data are too preliminary 61,5%, too little information 60.9%, anxious about unborn safety 68,3%		
Sezerol, 2023 [34]	hesitancy scale mean score 33.40 +/- 6.07	primary Education OR 0,52 (0,31-0,88); chronic disease 0,68 (0,36-1,29); vaccine advice 1,41 (0,98-2,012); Tetanus Vaccination Status (no) 3.68 (2,24-6,06); low Income Statue 1.59 (1,10-2,29)	62% fear of side effects; considered unnefcessary 18%; no trust in manufacturing companies 12.7%	
Simmons, 2022 [35	57%<br>Reasons: not enough knowledge about the vaccine 63%	younger age (aβ = 0.95, p = 0.025)<br>primiparity (aβ = 0.89, p = 0.624)<br>less urban context (aβ = 0.80, p = 0.041). etnicity not significantly associated; higer knowledge on vaccine (aβ= 0,82 p<0,001)		
Skirrow, 2022 [36]			safety concerns 61%; lack of safety data 46%; worries around the speed of vaccine development 17%	
Wang, 2022 [39]			74.4% harmful for the baby	Score for COVID- 19 knowledge–attitude–behavior 57.6±7.3 among non-vaccinated and 62.4±9.7 among vaccinated

a: adjusted; β: beta coefficient; HR: hazard ratio; OR: odds ratio, p: p-value; PR: prevalence ratio; r: spearman correlation; y: years
